# Crystal structures of (η^4^-cyclo­octa-1,5-diene)bis(1,3-di­methyl­imidazol-2-yl­idene)iridium(I) iodide and (η^4^-cyclo­octa-1,5-diene)bis­(1,3-di­ethyl­imidazol-2-yl­idene)iridium(I) iodide

**DOI:** 10.1107/S2056989020004235

**Published:** 2020-04-03

**Authors:** Chad M. Bernier, Christine M. DuChane, Joseph S. Merola

**Affiliations:** a Department of Chemistry, Virginia Tech, Blacksburg, VA 24061, USA

**Keywords:** crystal structure, N-heterocyclic carbene, iridium

## Abstract

The title complexes, (η^4^-cyclo­octa-1,5-diene)bis­(1,3-di­methyl­imidazol-2-yl­idene)iridium(I) iodide, [Ir(C_5_H_8_N_2_)_2_(C_8_H_12_)]I, (**1**) and (η^4^-cyclo­octa-1,5-di­ene)bis­(1,3-di­ethyl­imidazol-2-yl­idene)iridium(I) iodide, [Ir(C_7_H_12_N_2_)_2_(C_8_H_12_)]I, (**2**), were prepared using a modified literature method. After carrying out the oxidative addition of the amino acid l-proline to [Ir(COD)(IMe)_2_]I in water and slowly cooling the reaction to room temperature, a suitable crystal of **1** was obtained and analyzed by single-crystal X-ray diffraction at 100 K. Although this crystal structure has previously been reported in the *Pbam* space group, it was highly disordered and precise atomic coordinates were not calculated. A single crystal of **2** was also obtained by heating the complex in water and letting it slowly cool to room temperature. Complex **1** was found to crystallize in the monoclinic space group *C*2/*m*, while **2** crystallizes in the ortho­rhom­bic space group *Pccn*, both with *Z* = 4.

## Abstract 2

(η^4^-Cyclo­octa-1,5-diene)bis­(1,3-di­methyl­imidazol-2-yl­idene)iridium(I) iodide and (η^4^-cyclo­octa-1,5-diene)bis­(1,3-di­ethyl­imidazol-2-yl­idene)iridium(I) iodide were prepared using a modified literature method and crystallized from water in the monoclinic space group *C*2/*m* and the ortho­rhom­bic space group *Pccn*, respectively.

## Chemical context   

The Merola group has been inter­ested in the chemistry of electron-rich iridium compounds for many years (Frazier & Merola, 1992 ▸; Ladipo *et al.*, 1993 ▸; Selnau & Merola, 1993 ▸; Merola & Franks, 2013 ▸). Recently, we have begun examining the reactivity and catalytic applications of Ir^I^ N-heterocyclic carbene (NHC) complexes, which have previously been utilized for various transformations including hydrogenation (Hillier *et al.*, 2001 ▸), hydro­silylation (Viciano *et al.*, 2006 ▸), hydro­amination (Sipos *et al.*, 2016 ▸), H/D exchange (Cochrane *et al.*, 2014 ▸), and C—H bond functionalization (Frey *et al.*, 2006 ▸). While investigating the oxidative addition of amino acids to (η^4^-cyclo­octa-1,5-diene)bis­(1,3-di­methyl­imidazol-2-yl­idene)iridium(I) iodide in aqueous solution, cooling the reaction to room temperature yielded single crystals of the starting material [Ir(COD)(IMe)_2_]I, where IMe = 1,3-di­methyl­imidazol-2-yl­idene. Though Herrmann and coworkers previously described the crystal structure of this complex in the space group *Pbam* (Frey *et al.*, 2006 ▸), the anisotropic displacement parameters of the COD ligand were highly disordered; thus precise atomic coordinates could not be calculated. In an effort to advance the study of the structural properties and reactivity of Ir^I^ NHC complexes, we hereby report the single-crystal structure determination of (η^4^-cyclo­octa-1,5-diene)bis­(1,3-di­methyl­imidazol-2-yl­idene)iridium(I) iodide (**1**) and (η^4^-cyclo­octa-1,5-diene)bis­(1,3-di­ethyl­imidazol-2-yl­idene)iridium(I) iodide (**2**).
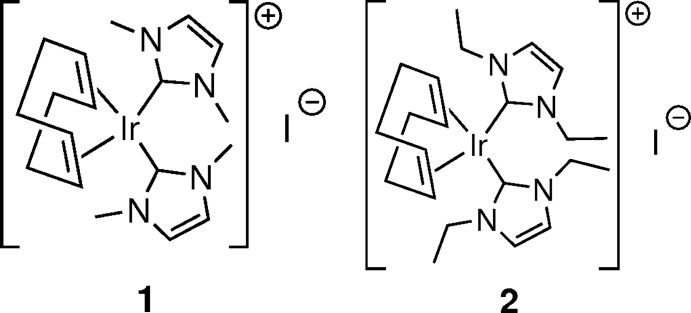


## Structural commentary   

**Figure 1:**
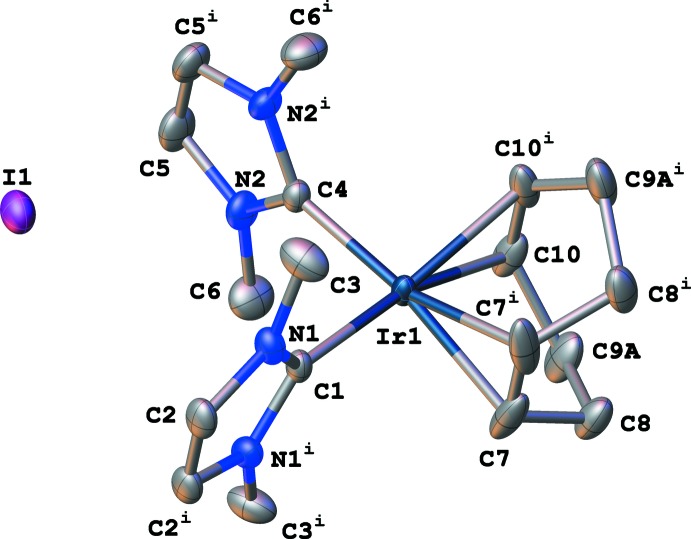
Displacement ellipsoid plot (50% probability) of (η^4^-cyclo­octa-1,5-diene)bis­(1,3-di­methyl­imidazol-2-yl­idene)iridium(I) iodide (**1**), showing part 1 of the disorder for the CH_2_ carbon atoms of the COD ring. Symmetry code: (i) *x*, 1 − *y*, *z*.

Complex **1** (CCDC ref code 1983640) crystallizes in the monoclinic space group *C*2/*m* with *Z* = 4 (Figs. 1 ▸ and 2 ▸), which differs from Herrmann’s original report of the ortho­rhom­bic space group *Pbam*. Ir1, C1, C4, and I1 lie in special positions on the mirror plane. The geometry around the metal center is nearly square planar, with the largest angle [C1—Ir1—C4 = 93.14 (10)°] and smallest angle [C7—Ir1—C10 (centroids) = 86.20°] having deviations of 3.14 and 3.80°, respectively, from the ideal 90° geometry. The average Ir—NHC bond length is 2.044 Å [Ir1—C1 = 2.037 (2), Ir1—C4 = 2.051 (2) Å] and the average Ir—C_COD_ bond length is 2.169 Å [Ir1—C7 = 2.163 (2) Å; Ir1—C10 = 2.174 (2) Å] with an Ir—COD_centroid_ distance of 2.047 Å, related by symmetry.

Complex **2** (CCDC ref code 1986045) crystallizes in the ortho­rhom­bic space group *Pccn* with *Z* = 4 (Fig. 3 ▸). Atom Ir1 lies in a special position on the twofold rotation axis. Similarly to **1**, the geometry around the metal center is nearly square planar, with the largest angle [C1—Ir1—C1 = 92.93 (12)°] and smallest angle [C8—Ir1—C9 (centroids) = 86.06°] having deviations of 2.92 and 3.94°, respectively, from the ideal 90° geometry. The Ir—NHC bond lengths [2.043 (2) Å] are related by symmetry. The average Ir—C_COD_ bond length is 2.172 Å [Ir1—C8 = 2.197 (2), Ir1—C9 = 2.147 (2) Å] with an Ir—COD_centroid_ distance of 2.058 Å, again related by symmetry.

This discrepancy in Ir—C_COD_ bond lengths and Ir—COD_centroid_ distances between the two complexes is likely due to the conformation of the COD ligand, which is a boat in **1** and a twist-boat in **2**.

## Supra­molecular features   

An examination of the packing diagrams for both title complexes show no unusual supra­molecular features.

## Database survey   

In our search for the COD bis-NHC moiety, we were somewhat surprised to find only ten reported IrCOD structures in the Cambridge Structural Database (CSD2019, update 3; Groom *et al.*, 2016 ▸) with two monodentate NHCs, including the original disordered structure reported by Herrmann (WEXKOA; Frey *et al.*, 2006 ▸). Structures similar to the title compound include a square-planar [(COD)bis­(1-ethyl-3-methyl­imidazol-2-yl­idene)iridium(I)] complex (BAHZER; Hinter­mair *et al.*, 2011 ▸) and a complex containing quinoline-functionalized NHC ligands (ROWWUX; Jiménez *et al.*, 2015 ▸), both in space group *P*21/*c* (No. 14). Other closely related structures include an iridium COD complex with pyrazolyl-functionalized NHC ligands (CEMVIA; Messerle *et al.*, 2006 ▸), and an iridium COD complex with penta­fluoro­benzyl functionalized NHCs (TESGEE; Burling *et al.*, 2006 ▸), both of which crystallized in space group *C*2/*c* (No. 15).

## Synthesis and crystallization   

The title compounds were synthesized using a modified literature procedure (Köcher & Herrmann, 1997 ▸). [Ir(COD)Cl]_2_ (500 mg, 0.744 mmol) and a magnetic stir bar were added to a flame-dried, nitro­gen-purged 100 mL Schlenk flask. Ethanol (20 mL) was added *via* syringe and the red solution was stirred. After 5 minutes, a solution of NaOEt in ethanol (1 *M*, 3.5 mL, 3.50 mmol) was added to the reaction flask dropwise. The solution was stirred for 1 h while the color slowly changed from red to bright yellow, indicating the formation of [Ir(COD)(OEt)]_2_. The NHC precursor 1,3-di­methyl­imidazolium iodide (840 mg, 3.75 mmol) or 1,3-di­ethyl­imidazolium iodide (945 mg, 3.75 mmol) was dissolved in ethanol (10 mL) and added to the stirring mixture *via* syringe. After 48 h, the bright-orange mixture was filtered through celite. The solvent was removed by rotary evaporation, and the residue was dissolved in minimal di­chloro­methane.

The crude product was purified *via* column chromatography with silica gel, first using a 1:1 mixture of cyclo­hexane to ethyl acetate as the mobile phase to collect the bright-yellow iridium mono-NHC complex, followed by 7% methanol in di­chloro­methane to collect the desired orange iridium bis-NHC product. The solvent was removed by rotary evaporation and the bright-orange solid was dried overnight under vacuum (449 mg, 49% for **1**; 415 mg, 42% for **2**). The products were characterized by ^1^H and ^13^C NMR spectroscopy in agreement with previously reported data.

Single crystals of **1** for X-ray crystallography were collected from a subsequent oxidative addition reaction. The title compound, l-proline, and 10 mL of water were added to a 6 dram vial and stirred overnight at 323 K. Upon slowly cooling the reaction mixture to room temperature, bright-orange crystals of the title compound grew and were collected. Single crystals of **2** were grown by dissolving the complex in water, heating it to 323 K, and letting the solution slowly cool to room temperature.

## Refinement   

Crystal data, data collection and structure refinement details are summarized in Table 1 ▸.

**Figure 2:**
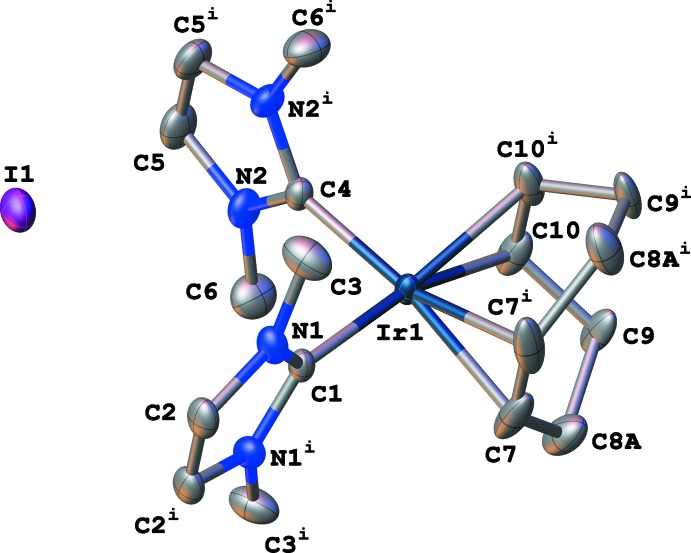
Displacement ellipsoid plot (50% probability) of (η^4^-cyclo­octa-1,5-diene)bis­(1,3-di­methyl­imidazol-2-yl­idene)iridium(I) iodide (**1**), showing part 2 of the disorder for the CH_2_ carbon atoms of the COD ring. Symmetry code: (i) *x*, 1 − *y*, *z*.

**Figure 3:**
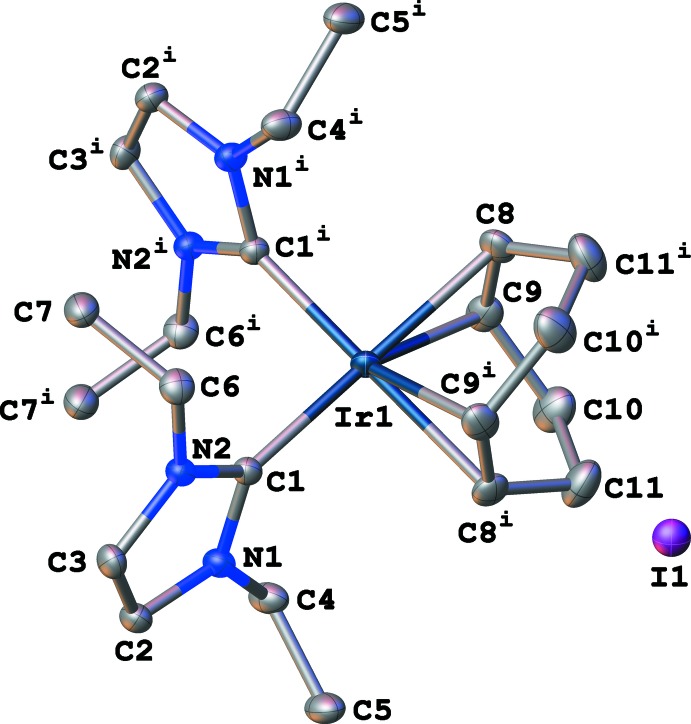
Displacement ellipsoid plot (50% probability) of (η^4^-cyclo­octa-1,5-diene)bis­(1,3-di­ethyl­imidazol-2-yl­idene)iridium(I) iodide (**2**).Symmetry code: (i) 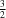 − *x*, 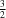 − *y*, *z*.

Compound **1** was solved with *SHELXS* and refined with *SHELXL* within *OLEX2*. The refinement proceeded quite well although the displacement ellipsoids for the CH_2_ carbon atoms of the COD ring were overly elongated, suggesting that there was possible disorder. In *OLEX2*, the disorder tools were utilized to split the carbon atoms while adding *SHELXL* SIMU restraint. The disorder model appeared to refine well with reasonable displacement ellipsoids. Fig. 1 ▸ shows part 1 of the disorder and Fig. 2 ▸ shows part 2. Both parts show nearly equal occupancies refining to 0.515 (19):0.485 (19). The two parts seem best described as the result of static disorder wherein the saturated portion of the COD ring is slightly twisted. The unsaturated carbon atoms are also likely a part of the disorder, but the positional change is so slight as to not warrant (and to resist) modeling. However, a consequence of this slight disorder is that generating the entire mol­ecule does generate two different hydrogen-atom positions, also refining to 0.515 (19):0.485 (19) relative occupancies.

Data reduction, solution and refinement for **2** presented some inter­esting issues that are discussed here. The data were collected on a XtaLAB Synergy, Dualflex, HyPix diffractometer. Data reduction was performed with *CrysAlisPro171.40_64.67a* (Rigaku OD, 2018 ▸). The crystal was of good quality and peak searching found 9425 peaks that were merged to 5446 profiles. Unit-cell calculations fit 98.2% of the peaks to the cell 9.1397 (5), 10.6193 (7), 12.3249 (6), 89.980 (5). 89.988 (4), 89.965 (6). Further refinement and space group determination led to the finalization of the data in ortho­rhom­bic *P. SHELXT* within *OLEX2* was used for structure solution and several non-centrosymmetric space groups were identified with nearly equal figure of merit. Attempts were made to refine the structure in all five of the proffered space groups and the only one that provided a reasonable solution was *P*2_1_2_1_2. However, while the structure refinement parameters were ‘reasonable’, several displacement ellipsoids in the finalized model were elongated along strange directions. The data were reexamined and a close view of the Ewald sphere showed weak, but clearly present peaks between the axes. The ∼9 Å axis was doubled and now all peaks were aligned fully with the new axes of 18.2790 (10), 10.6196 (7), 12.3245 (6), 89.979 (5), 89.985 (4), 89.965 (5). With those particular settings in *CrysAlis*, the only reasonable unit cell found was triclinic.

Moving into *OLEX2* again, a solution was found in *P* 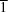 that refined into a solution with excellent figures of merit and well-shaped displacement ellipsoids with *Z* = 4. However, it was noted that the heavy atoms, iridium and iodine all had coordinates that suggested they sat on special positions, *e.g. x* = 0.7500. ADDSYM in *PLATON* (Spek, 2020 ▸) was used to search for higher symmetries and the result suggested that *Pccn* was an appropriate high-symmetry space group. The newly created data and instruction files from *PLATON* were used in *OLEX2* and the structure in *Pccn* solved and refined cleanly into the final structure. With this result in hand, the raw data were re-reduced, the originally found *x* axis was again doubled and space-group analysis was re-performed with slightly larger angle tolerances (0.03 *vs* 0.015). *Pccn* was then clearly identified as the top match for the space group. The data and instruction files were once more used in *OLEX2* and *SHELXT* used as the solution program, which determined that *Pccn* was the best space group. Refinement led to the final structure solution reported in this paper.

## Supplementary Material

Crystal structure: contains datablock(s) 1, 2, New_Global_Publ_Block. DOI: 10.1107/S2056989020004235/mw2156sup1.cif

Structure factors: contains datablock(s) 1. DOI: 10.1107/S2056989020004235/mw21561sup2.hkl

Supporting information file. DOI: 10.1107/S2056989020004235/mw21561sup4.mol

Structure factors: contains datablock(s) 2. DOI: 10.1107/S2056989020004235/mw21562sup3.hkl

Supporting information file. DOI: 10.1107/S2056989020004235/mw21562sup5.mol

CCDC references: 1986045, 1983640

Additional supporting information:  crystallographic information; 3D view; checkCIF report

## Figures and Tables

**Table 1:** Experimental details

	**1**	**2**
Crystal data		
Chemical formula	[Ir(C_5_H_8_N_2_)_2_(C_8_H_12_)]I	[Ir(C_7_H_12_N_2_)_2_(C_8_H_12_)]I
*M*_r_	619.54	675.65
Crystal system, space group	Monoclinic, *C*2/*m*	Orthorhombic, *P* *c* *c* *n*
Temperature (K)	100	100
*a*, *b*, *c* (Å)	26.6519 (4), 8.3070 (2), 9.7852 (2)	10.6041 (2), 12.3058 (2), 18.2513 (3)
α, β, γ (°)	90, 100.241 (2), 90	90, 90, 90
*V* (Å^3^)	2131.90 (8)	2381.65 (7)
*Z*	4	4
Radiation type	Mo *K*α	Mo *K*α
μ (mm^−1^)	7.72	6.92
Crystal size (mm)	0.54 × 0.22 × 0.11	0.38 × 0.17 × 0.12

Data collection		
Diffractometer	XtaLAB Synergy, Dualflex, HyPix	XtaLAB Synergy, Dualflex, HyPix
Absorption correction	Gaussian (*CrysAlis PRO*;Rigaku OD, 2018 ▸)	Gaussian (*CrysAlis PRO*;Rigaku OD, 2018 ▸)
*T*_min_, *T*_max_	0.179, 0.960	0.318, 1.000
No. of measured, independent and observed [*I* > 2σ(*I*)] reflections	27006, 5884, 5415	59059, 6352, 3839
*R*_int_	0.034	0.062
(sin θ/λ)_max_ (Å^−1^)	0.871	0.870

Refinement		
*R*[*F* ^2^ > 2σ(*F* ^2^)], *wR*(*F* ^2^), *S*	0.024, 0.059, 1.04	0.030, 0.060, 1.00
No. of reflections	5884	6352
No. of parameters	136	130
No. of restraints	12	0
H-atom treatment	H-atom parameters constrained	H-atom parameters constrained
Δρ_max_, Δρ_min_ (e Å^−3^)	2.40, −1.29	1.54, −0.83

Computer programs: *CrysAlis PRO* (Rigaku OD, 2018 ▸), *SHELXT* (Sheldrick, 2015*a* ▸), *SHELXL* (Sheldrick, 2015*b* ▸) and *OLEX2* (Dolomanov *et al.*, 2009 ▸).

## supplementary crystallographic information

### (η4-Cycloocta-1,5-diene)bis(1,3-dimethylimidazol-2-ylidene)iridium(I) iodide (1) Crystal data

**Table:**

[Ir(C_5_H_8_N_2_)_2_(C_8_H_12_)]I	*F*(000) = 1176
*M**_r_* = 619.54	*D*_x_ = 1.930 Mg m^−^^3^
Monoclinic, *C*2/*m*	Mo *K*α radiation, λ = 0.71073 Å
*a* = 26.6519 (4) Å	Cell parameters from 17811 reflections
*b* = 8.3070 (2) Å	θ = 2.6–38.3°
*c* = 9.7852 (2) Å	µ = 7.72 mm^−^^1^
β = 100.241 (2)°	*T* = 100 K
*V* = 2131.90 (8) Å^3^	Prism, orange
*Z* = 4	0.54 × 0.22 × 0.11 mm

### (η4-Cycloocta-1,5-diene)bis(1,3-dimethylimidazol-2-ylidene)iridium(I) iodide (1) Data collection

**Table:**

XtaLAB Synergy, Dualflex, HyPix diffractometer	5884 independent reflections
Radiation source: micro-focus sealed X-ray tube, PhotonJet (Mo) X-ray Source	5415 reflections with *I* > 2σ(*I*)
Mirror monochromator	*R*_int_ = 0.034
ω scans	θ_max_ = 38.2°, θ_min_ = 3.1°
Absorption correction: gaussian (CrysAlisPro;Rigaku OD, 2018)	*h* = −45→44
*T*_min_ = 0.179, *T*_max_ = 0.960	*k* = −14→13
27006 measured reflections	*l* = −16→16

### (η4-Cycloocta-1,5-diene)bis(1,3-dimethylimidazol-2-ylidene)iridium(I) iodide (1) Refinement

**Table:**

Refinement on *F*^2^	12 restraints
Least-squares matrix: full	Hydrogen site location: inferred from neighbouring sites
*R*[*F*^2^ > 2σ(*F*^2^)] = 0.024	H-atom parameters constrained
*wR*(*F*^2^) = 0.059	*w* = 1/[σ^2^(*F*_o_^2^) + (0.0317*P*)^2^ + 1.4859*P*] where *P* = (*F*_o_^2^ + 2*F*_c_^2^)/3
*S* = 1.04	(Δ/σ)_max_ = 0.002
5884 reflections	Δρ_max_ = 2.40 e Å^−^^3^
136 parameters	Δρ_min_ = −1.29 e Å^−^^3^

### (η4-Cycloocta-1,5-diene)bis(1,3-dimethylimidazol-2-ylidene)iridium(I) iodide (1) Special details

**Table:**

Geometry. All esds (except the esd in the dihedral angle between two l.s. planes) are estimated using the full covariance matrix. The cell esds are taken into account individually in the estimation of esds in distances, angles and torsion angles; correlations between esds in cell parameters are only used when they are defined by crystal symmetry. An approximate (isotropic) treatment of cell esds is used for estimating esds involving l.s. planes.

### (η4-Cycloocta-1,5-diene)bis(1,3-dimethylimidazol-2-ylidene)iridium(I) iodide (1) Fractional atomic coordinates and isotropic or equivalent isotropic displacement parameters (Å^2^)

**Table:**

	*x*	*y*	*z*	*U*_iso_*/*U*_eq_	Occ. (<1)
Ir1	0.35715 (2)	0.500000	0.71082 (2)	0.01615 (3)	
N1	0.36047 (6)	0.6287 (2)	0.41903 (16)	0.0216 (3)	
N2	0.46647 (6)	0.3712 (2)	0.79484 (18)	0.0242 (3)	
C1	0.36077 (9)	0.500000	0.5047 (2)	0.0184 (4)	
C2	0.36009 (8)	0.5804 (3)	0.28291 (19)	0.0254 (4)	
H2	0.359874	0.648608	0.204874	0.031*	
C3	0.36109 (11)	0.7956 (3)	0.4619 (2)	0.0346 (5)	
H3A	0.394582	0.842411	0.458346	0.052*	
H3B	0.354380	0.802066	0.557045	0.052*	
H3C	0.334734	0.855247	0.399422	0.052*	
C4	0.43494 (9)	0.500000	0.7697 (3)	0.0189 (4)	
C5	0.51693 (8)	0.4201 (3)	0.8329 (2)	0.0336 (5)	
H5	0.545889	0.352001	0.854780	0.040*	
C6	0.45066 (10)	0.2032 (3)	0.7875 (3)	0.0353 (5)	
H6A	0.463915	0.149514	0.712161	0.053*	
H6B	0.413344	0.197190	0.769749	0.053*	
H6C	0.464082	0.149835	0.875787	0.053*	
C7	0.27918 (8)	0.4158 (4)	0.6632 (2)	0.0436 (7)	
H7A	0.268531	0.362122	0.571156	0.052*	0.485 (19)
H7B	0.270988	0.372499	0.566457	0.052*	0.515 (19)
C8	0.2597 (3)	0.3445 (15)	0.7821 (6)	0.0346 (18)	0.485 (19)
H8A	0.237419	0.251494	0.749983	0.041*	0.485 (19)
H8B	0.239134	0.425165	0.822295	0.041*	0.485 (19)
C9	0.2980 (3)	0.3377 (15)	0.9218 (8)	0.0330 (18)	0.485 (19)
H9A	0.274812	0.415708	0.955269	0.040*	0.485 (19)
H9B	0.303203	0.246025	0.987461	0.040*	0.485 (19)
C10	0.34831 (8)	0.4171 (3)	0.9159 (2)	0.0329 (5)	
H10	0.380731	0.371865	0.969269	0.040*	0.485 (19)
H10A	0.378957	0.364754	0.972248	0.040*	0.515 (19)
I1	0.59789 (2)	0.000000	0.87332 (2)	0.03089 (5)	
C9A	0.3044 (3)	0.2893 (16)	0.8926 (10)	0.0404 (19)	0.515 (19)
H9AA	0.317748	0.186061	0.863449	0.049*	0.515 (19)
H9AB	0.292215	0.270733	0.981216	0.049*	0.515 (19)
C8A	0.2740 (4)	0.2775 (14)	0.7760 (6)	0.0386 (18)	0.515 (19)
H8AA	0.237614	0.251443	0.773250	0.046*	0.515 (19)
H8AB	0.291638	0.178533	0.753713	0.046*	0.515 (19)

### (η4-Cycloocta-1,5-diene)bis(1,3-dimethylimidazol-2-ylidene)iridium(I) iodide (1) Atomic displacement parameters (Å^2^)

**Table:**

	*U*^11^	*U*^22^	*U*^33^	*U*^12^	*U*^13^	*U*^23^
Ir1	0.01272 (4)	0.02483 (5)	0.01113 (4)	0.000	0.00271 (2)	0.000
N1	0.0225 (7)	0.0278 (8)	0.0148 (6)	0.0037 (6)	0.0047 (5)	0.0019 (5)
N2	0.0164 (6)	0.0314 (9)	0.0245 (7)	0.0044 (6)	0.0025 (5)	0.0026 (6)
C1	0.0153 (9)	0.0264 (12)	0.0142 (9)	0.000	0.0044 (7)	0.000
C2	0.0251 (8)	0.0384 (11)	0.0139 (6)	0.0012 (7)	0.0064 (6)	0.0023 (7)
C3	0.0534 (14)	0.0269 (10)	0.0228 (9)	0.0045 (10)	0.0050 (9)	0.0032 (8)
C4	0.0154 (9)	0.0261 (12)	0.0155 (9)	0.000	0.0036 (7)	0.000
C5	0.0168 (7)	0.0498 (14)	0.0334 (11)	0.0050 (8)	0.0019 (7)	0.0024 (9)
C6	0.0324 (11)	0.0291 (11)	0.0430 (13)	0.0065 (9)	0.0032 (9)	0.0033 (9)
C7	0.0195 (8)	0.092 (2)	0.0185 (8)	−0.0201 (11)	0.0007 (6)	0.0036 (10)
C8	0.022 (2)	0.057 (5)	0.024 (2)	−0.012 (3)	0.0043 (17)	0.005 (3)
C9	0.022 (3)	0.063 (5)	0.014 (2)	−0.014 (3)	0.0043 (16)	0.009 (2)
C10	0.0205 (8)	0.0624 (15)	0.0154 (7)	−0.0086 (9)	0.0018 (6)	0.0091 (8)
I1	0.03408 (10)	0.03389 (10)	0.02760 (9)	0.000	0.01334 (7)	0.000
C9A	0.029 (3)	0.069 (6)	0.025 (3)	−0.011 (3)	0.009 (2)	0.012 (3)
C8A	0.034 (3)	0.055 (5)	0.027 (2)	−0.019 (3)	0.006 (2)	0.004 (2)

### (η4-Cycloocta-1,5-diene)bis(1,3-dimethylimidazol-2-ylidene)iridium(I) iodide (1) Geometric parameters (Å, º)

**Table:**

Ir1—C1	2.037 (2)	C6—H6C	0.9800
Ir1—C4	2.051 (2)	C7—C7^i^	1.399 (7)
Ir1—C7^i^	2.163 (2)	C7—H7A	1.0000
Ir1—C7	2.163 (2)	C7—H7B	1.0000
Ir1—C10^i^	2.174 (2)	C7—C8	1.480 (6)
Ir1—C10	2.174 (2)	C7—C8A	1.616 (8)
N1—C1	1.357 (2)	C8—H8A	0.9900
N1—C2	1.389 (2)	C8—H8B	0.9900
N1—C3	1.448 (3)	C8—C9A	1.530 (12)
N2—C4	1.356 (2)	C9—H9A	0.9900
N2—C5	1.391 (3)	C9—H9B	0.9900
N2—C6	1.456 (3)	C9—C10	1.505 (8)
C2—C2^i^	1.336 (5)	C9—C8A	1.540 (10)
C2—H2	0.9500	C10—C10^i^	1.378 (6)
C3—H3A	0.9800	C10—H10	1.0000
C3—H3B	0.9800	C10—H10A	1.0000
C3—H3C	0.9800	C10—C9A	1.567 (10)
C5—C5^i^	1.328 (5)	C9A—H9AA	0.9900
C5—H5	0.9500	C9A—H9AB	0.9900
C6—H6A	0.9800	C8A—H8AA	0.9900
C6—H6B	0.9800	C8A—H8AB	0.9900
			
C1—Ir1—C4	93.14 (10)	Ir1—C7—H7B	111.1
C1—Ir1—C7	89.97 (9)	C7^i^—C7—Ir1	71.14 (10)
C1—Ir1—C7^i^	89.97 (9)	C7^i^—C7—H7A	116.5
C1—Ir1—C10	161.15 (8)	C7^i^—C7—H7B	111.1
C1—Ir1—C10^i^	161.16 (8)	C7^i^—C7—C8	113.6 (5)
C4—Ir1—C7^i^	160.90 (9)	C7^i^—C7—C8A	135.3 (4)
C4—Ir1—C7	160.90 (9)	C8—C7—Ir1	114.8 (3)
C4—Ir1—C10	90.61 (8)	C8—C7—H7A	116.5
C4—Ir1—C10^i^	90.61 (8)	C8A—C7—Ir1	106.1 (3)
C7^i^—Ir1—C7	37.71 (19)	C8A—C7—H7B	111.1
C7—Ir1—C10^i^	92.50 (9)	C7—C8—H8A	109.7
C7^i^—Ir1—C10	92.49 (9)	C7—C8—H8B	109.7
C7^i^—Ir1—C10^i^	80.72 (8)	C7—C8—C9A	109.7 (5)
C7—Ir1—C10	80.72 (8)	H8A—C8—H8B	108.2
C10^i^—Ir1—C10	36.95 (15)	C9A—C8—H8A	109.7
C1—N1—C2	111.26 (18)	C9A—C8—H8B	109.7
C1—N1—C3	125.22 (17)	H9A—C9—H9B	108.2
C2—N1—C3	123.51 (18)	C10—C9—H9A	109.8
C4—N2—C5	110.93 (19)	C10—C9—H9B	109.8
C4—N2—C6	125.66 (17)	C10—C9—C8A	109.4 (5)
C5—N2—C6	123.40 (19)	C8A—C9—H9A	109.8
N1^i^—C1—Ir1	127.97 (11)	C8A—C9—H9B	109.8
N1—C1—Ir1	127.97 (11)	Ir1—C10—H10	112.1
N1—C1—N1^i^	103.9 (2)	Ir1—C10—H10A	115.8
N1—C2—H2	126.6	C9—C10—Ir1	114.7 (3)
C2^i^—C2—N1	106.78 (12)	C9—C10—H10A	115.8
C2^i^—C2—H2	126.6	C10^i^—C10—Ir1	71.53 (8)
N1—C3—H3A	109.5	C10^i^—C10—C9	116.0 (5)
N1—C3—H3B	109.5	C10^i^—C10—H10	112.1
N1—C3—H3C	109.5	C10^i^—C10—H10A	115.8
H3A—C3—H3B	109.5	C10^i^—C10—C9A	132.7 (5)
H3A—C3—H3C	109.5	C9A—C10—Ir1	106.3 (4)
H3B—C3—H3C	109.5	C9A—C10—H10	112.1
N2—C4—Ir1	127.89 (11)	C8—C9A—C10	111.6 (7)
N2^i^—C4—Ir1	127.89 (11)	C8—C9A—H9AA	109.3
N2^i^—C4—N2	104.2 (2)	C8—C9A—H9AB	109.3
N2—C5—H5	126.5	C10—C9A—H9AA	109.3
C5^i^—C5—N2	106.96 (13)	C10—C9A—H9AB	109.3
C5^i^—C5—H5	126.5	H9AA—C9A—H9AB	108.0
N2—C6—H6A	109.5	C7—C8A—H8AA	109.8
N2—C6—H6B	109.5	C7—C8A—H8AB	109.8
N2—C6—H6C	109.5	C9—C8A—C7	109.3 (6)
H6A—C6—H6B	109.5	C9—C8A—H8AA	109.8
H6A—C6—H6C	109.5	C9—C8A—H8AB	109.8
H6B—C6—H6C	109.5	H8AA—C8A—H8AB	108.3
Ir1—C7—H7A	116.5		
			
Ir1—C7—C8—C9A	−21.8 (8)	C5—N2—C4—N2^i^	−0.9 (3)
Ir1—C7—C8A—C9	46.8 (7)	C6—N2—C4—Ir1	−1.4 (3)
Ir1—C10—C9A—C8	−43.0 (7)	C6—N2—C4—N2^i^	177.51 (16)
C1—N1—C2—C2^i^	0.08 (17)	C6—N2—C5—C5^i^	−177.88 (18)
C2—N1—C1—Ir1	−176.04 (16)	C7^i^—C7—C8—C9A	−101.0 (6)
C2—N1—C1—N1^i^	−0.1 (3)	C7^i^—C7—C8A—C9	−32.5 (9)
C3—N1—C1—Ir1	4.7 (3)	C7—C8—C9A—C10	43.1 (7)
C3—N1—C1—N1^i^	−179.40 (16)	C10—C9—C8A—C7	−44.8 (8)
C3—N1—C2—C2^i^	179.37 (17)	C10^i^—C10—C9A—C8	36.7 (8)
C4—N2—C5—C5^i^	0.58 (19)	C8A—C9—C10—Ir1	21.6 (8)
C5—N2—C4—Ir1	−179.85 (17)	C8A—C9—C10—C10^i^	102.2 (6)

Symmetry code: (i) *x*, −*y*+1, *z*.

### (η4-Cycloocta-1,5-diene)bis(1,3-diethylimidazol-2-ylidene)iridium(I) iodide (2) Crystal data

**Table:**

[Ir(C_7_H_12_N_2_)_2_(C_8_H_12_)]I	*D*_x_ = 1.884 Mg m^−^^3^
*M**_r_* = 675.65	Mo *K*α radiation, λ = 0.71073 Å
Orthorhombic, *P**c**c**n*	Cell parameters from 17338 reflections
*a* = 10.6041 (2) Å	θ = 2.7–37.9°
*b* = 12.3058 (2) Å	µ = 6.92 mm^−^^1^
*c* = 18.2513 (3) Å	*T* = 100 K
*V* = 2381.65 (7) Å^3^	Block, orange
*Z* = 4	0.38 × 0.17 × 0.12 mm
*F*(000) = 1304	

### (η4-Cycloocta-1,5-diene)bis(1,3-diethylimidazol-2-ylidene)iridium(I) iodide (2) Data collection

**Table:**

XtaLAB Synergy, Dualflex, HyPix diffractometer	6352 independent reflections
Radiation source: micro-focus sealed X-ray tube, PhotonJet (Mo) X-ray Source	3839 reflections with *I* > 2σ(*I*)
Mirror monochromator	*R*_int_ = 0.062
Detector resolution: 10.0000 pixels mm^-1^	θ_max_ = 38.2°, θ_min_ = 2.5°
ω scans	*h* = −16→17
Absorption correction: gaussian (CrysAlisPro;Rigaku OD, 2018)	*k* = −21→21
*T*_min_ = 0.318, *T*_max_ = 1.000	*l* = −30→31
59059 measured reflections	

### (η4-Cycloocta-1,5-diene)bis(1,3-diethylimidazol-2-ylidene)iridium(I) iodide (2) Refinement

**Table:**

Refinement on *F*^2^	0 restraints
Least-squares matrix: full	Hydrogen site location: inferred from neighbouring sites
*R*[*F*^2^ > 2σ(*F*^2^)] = 0.030	H-atom parameters constrained
*wR*(*F*^2^) = 0.060	*w* = 1/[σ^2^(*F*_o_^2^) + (0.022*P*)^2^ + 1.4351*P*] where *P* = (*F*_o_^2^ + 2*F*_c_^2^)/3
*S* = 1.00	(Δ/σ)_max_ = 0.002
6352 reflections	Δρ_max_ = 1.54 e Å^−^^3^
130 parameters	Δρ_min_ = −0.83 e Å^−^^3^

### (η4-Cycloocta-1,5-diene)bis(1,3-diethylimidazol-2-ylidene)iridium(I) iodide (2) Special details

**Table:**

Geometry. All esds (except the esd in the dihedral angle between two l.s. planes) are estimated using the full covariance matrix. The cell esds are taken into account individually in the estimation of esds in distances, angles and torsion angles; correlations between esds in cell parameters are only used when they are defined by crystal symmetry. An approximate (isotropic) treatment of cell esds is used for estimating esds involving l.s. planes.

### (η4-Cycloocta-1,5-diene)bis(1,3-diethylimidazol-2-ylidene)iridium(I) iodide (2) Fractional atomic coordinates and isotropic or equivalent isotropic displacement parameters (Å^2^)

**Table:**

	*x*	*y*	*z*	*U*_iso_*/*U*_eq_	
Ir1	0.750000	0.750000	0.50409 (2)	0.01428 (3)	
N1	0.66768 (19)	0.56037 (15)	0.40954 (10)	0.0170 (4)	
N2	0.5533 (2)	0.69802 (15)	0.38262 (10)	0.0160 (4)	
C1	0.6489 (2)	0.66696 (18)	0.42698 (11)	0.0147 (4)	
C2	0.5863 (2)	0.52747 (19)	0.35491 (12)	0.0197 (5)	
H2	0.582250	0.458717	0.333882	0.024*	
C3	0.5145 (2)	0.61300 (19)	0.33789 (13)	0.0193 (4)	
H3	0.450781	0.615208	0.302881	0.023*	
C4	0.7633 (2)	0.49015 (18)	0.44314 (14)	0.0217 (5)	
H4A	0.812864	0.532569	0.477466	0.026*	
H4B	0.819765	0.463618	0.405332	0.026*	
C5	0.7056 (3)	0.3938 (2)	0.48314 (15)	0.0258 (5)	
H5A	0.666738	0.345931	0.448297	0.039*	
H5B	0.643277	0.419285	0.517187	0.039*	
H5C	0.770442	0.355409	0.509178	0.039*	
C6	0.5011 (2)	0.80817 (19)	0.37857 (13)	0.0197 (5)	
H6A	0.521838	0.847322	0.423096	0.024*	
H6B	0.409999	0.804105	0.374896	0.024*	
C7	0.5529 (3)	0.86951 (19)	0.31300 (13)	0.0238 (5)	
H7A	0.531961	0.831132	0.268855	0.036*	
H7B	0.642915	0.875246	0.317200	0.036*	
H7C	0.516639	0.940945	0.311475	0.036*	
C8	0.8334 (2)	0.8517 (2)	0.59035 (13)	0.0218 (5)	
H8	0.877875	0.916010	0.572020	0.026*	
C9	0.9006 (2)	0.7551 (2)	0.58265 (13)	0.0232 (4)	
H9	0.983639	0.765350	0.560128	0.028*	
C10	0.8939 (3)	0.6559 (2)	0.63123 (15)	0.0317 (6)	
H10A	0.940977	0.669868	0.675826	0.038*	
H10B	0.933711	0.595331	0.606313	0.038*	
C11	0.7595 (3)	0.6247 (2)	0.65118 (15)	0.0329 (6)	
H11A	0.756815	0.547796	0.662661	0.039*	
H11B	0.734462	0.664384	0.694739	0.039*	
I1	0.250000	0.750000	0.71451 (2)	0.02175 (4)	

### (η4-Cycloocta-1,5-diene)bis(1,3-diethylimidazol-2-ylidene)iridium(I) iodide (2) Atomic displacement parameters (Å^2^)

**Table:**

	*U*^11^	*U*^22^	*U*^33^	*U*^12^	*U*^13^	*U*^23^
Ir1	0.01317 (5)	0.01477 (5)	0.01492 (5)	−0.00400 (6)	0.000	0.000
N1	0.0177 (10)	0.0156 (8)	0.0176 (9)	−0.0029 (7)	−0.0019 (7)	−0.0005 (7)
N2	0.0179 (10)	0.0145 (9)	0.0155 (8)	−0.0021 (7)	−0.0004 (8)	−0.0007 (7)
C1	0.0151 (10)	0.0148 (9)	0.0144 (9)	−0.0025 (7)	0.0000 (7)	−0.0004 (8)
C2	0.0237 (12)	0.0175 (10)	0.0178 (10)	−0.0052 (9)	−0.0024 (9)	−0.0011 (8)
C3	0.0213 (12)	0.0216 (11)	0.0149 (10)	−0.0029 (9)	−0.0039 (9)	−0.0012 (9)
C4	0.0212 (14)	0.0171 (9)	0.0267 (11)	−0.0022 (9)	−0.0066 (10)	0.0008 (8)
C5	0.0333 (14)	0.0170 (11)	0.0271 (13)	−0.0048 (10)	−0.0070 (11)	0.0032 (10)
C6	0.0194 (12)	0.0193 (11)	0.0205 (11)	0.0035 (9)	0.0006 (9)	−0.0013 (9)
C7	0.0325 (15)	0.0189 (11)	0.0200 (11)	−0.0005 (10)	−0.0016 (10)	0.0007 (9)
C8	0.0190 (12)	0.0247 (12)	0.0216 (11)	−0.0084 (9)	−0.0032 (9)	−0.0039 (10)
C9	0.0175 (9)	0.0292 (11)	0.0230 (10)	−0.0072 (12)	−0.0054 (8)	0.0031 (13)
C10	0.0270 (15)	0.0384 (15)	0.0297 (14)	−0.0029 (12)	−0.0084 (11)	0.0090 (12)
C11	0.0319 (16)	0.0418 (14)	0.0249 (11)	−0.0040 (14)	−0.0006 (13)	0.0124 (11)
I1	0.01710 (8)	0.02436 (9)	0.02378 (10)	−0.00028 (11)	0.000	0.000

### (η4-Cycloocta-1,5-diene)bis(1,3-diethylimidazol-2-ylidene)iridium(I) iodide (2) Geometric parameters (Å, º)

**Table:**

Ir1—C1^i^	2.043 (2)	C5—H5B	0.9600
Ir1—C1	2.043 (2)	C5—H5C	0.9600
Ir1—C8	2.197 (2)	C6—H6A	0.9700
Ir1—C8^i^	2.197 (2)	C6—H6B	0.9700
Ir1—C9^i^	2.147 (2)	C6—C7	1.518 (3)
Ir1—C9	2.147 (2)	C7—H7A	0.9600
N1—C1	1.364 (3)	C7—H7B	0.9600
N1—C2	1.379 (3)	C7—H7C	0.9600
N1—C4	1.467 (3)	C8—H8	0.9800
N2—C1	1.353 (3)	C8—C9	1.393 (4)
N2—C3	1.389 (3)	C8—C11^i^	1.513 (4)
N2—C6	1.466 (3)	C9—H9	0.9800
C2—H2	0.9300	C9—C10	1.510 (4)
C2—C3	1.336 (3)	C10—H10A	0.9700
C3—H3	0.9300	C10—H10B	0.9700
C4—H4A	0.9700	C10—C11	1.520 (4)
C4—H4B	0.9700	C11—H11A	0.9700
C4—C5	1.521 (3)	C11—H11B	0.9700
C5—H5A	0.9600		
			
C1^i^—Ir1—C1	92.93 (12)	H5A—C5—H5B	109.5
C1—Ir1—C8^i^	89.85 (9)	H5A—C5—H5C	109.5
C1^i^—Ir1—C8^i^	171.79 (9)	H5B—C5—H5C	109.5
C1—Ir1—C8	171.79 (9)	N2—C6—H6A	109.4
C1^i^—Ir1—C8	89.85 (9)	N2—C6—H6B	109.4
C1^i^—Ir1—C9^i^	149.88 (10)	N2—C6—C7	111.30 (19)
C1^i^—Ir1—C9	93.15 (9)	H6A—C6—H6B	108.0
C1—Ir1—C9	149.88 (10)	C7—C6—H6A	109.4
C1—Ir1—C9^i^	93.15 (9)	C7—C6—H6B	109.4
C8^i^—Ir1—C8	88.46 (13)	C6—C7—H7A	109.5
C9^i^—Ir1—C8	80.66 (9)	C6—C7—H7B	109.5
C9—Ir1—C8	37.37 (10)	C6—C7—H7C	109.5
C9^i^—Ir1—C8^i^	37.37 (10)	H7A—C7—H7B	109.5
C9—Ir1—C8^i^	80.66 (9)	H7A—C7—H7C	109.5
C9^i^—Ir1—C9	96.21 (13)	H7B—C7—H7C	109.5
C1—N1—C2	111.1 (2)	Ir1—C8—H8	114.1
C1—N1—C4	124.74 (19)	C9—C8—Ir1	69.36 (13)
C2—N1—C4	124.18 (19)	C9—C8—H8	114.1
C1—N2—C3	111.16 (19)	C9—C8—C11^i^	124.8 (2)
C1—N2—C6	125.05 (19)	C11^i^—C8—Ir1	111.88 (17)
C3—N2—C6	123.70 (19)	C11^i^—C8—H8	114.1
N1—C1—Ir1	124.39 (16)	Ir1—C9—H9	113.1
N2—C1—Ir1	131.63 (17)	C8—C9—Ir1	73.27 (14)
N2—C1—N1	103.98 (19)	C8—C9—H9	113.1
N1—C2—H2	126.5	C8—C9—C10	127.3 (2)
C3—C2—N1	107.1 (2)	C10—C9—Ir1	109.49 (17)
C3—C2—H2	126.5	C10—C9—H9	113.1
N2—C3—H3	126.6	C9—C10—H10A	109.0
C2—C3—N2	106.7 (2)	C9—C10—H10B	109.0
C2—C3—H3	126.6	C9—C10—C11	112.9 (2)
N1—C4—H4A	109.1	H10A—C10—H10B	107.8
N1—C4—H4B	109.1	C11—C10—H10A	109.0
N1—C4—C5	112.4 (2)	C11—C10—H10B	109.0
H4A—C4—H4B	107.8	C8^i^—C11—C10	112.7 (2)
C5—C4—H4A	109.1	C8^i^—C11—H11A	109.0
C5—C4—H4B	109.1	C8^i^—C11—H11B	109.0
C4—C5—H5A	109.5	C10—C11—H11A	109.0
C4—C5—H5B	109.5	C10—C11—H11B	109.0
C4—C5—H5C	109.5	H11A—C11—H11B	107.8
			
Ir1—C8—C9—C10	−102.1 (2)	C3—N2—C6—C7	−76.3 (3)
Ir1—C9—C10—C11	−39.1 (3)	C4—N1—C1—Ir1	−0.3 (3)
N1—C2—C3—N2	0.1 (3)	C4—N1—C1—N2	179.6 (2)
C1—N1—C2—C3	−0.6 (3)	C4—N1—C2—C3	−179.4 (2)
C1—N1—C4—C5	118.4 (2)	C6—N2—C1—Ir1	2.5 (3)
C1—N2—C3—C2	0.4 (3)	C6—N2—C1—N1	−177.47 (19)
C1—N2—C6—C7	100.0 (3)	C6—N2—C3—C2	177.2 (2)
C2—N1—C1—Ir1	−179.19 (16)	C8—C9—C10—C11	44.4 (4)
C2—N1—C1—N2	0.8 (3)	C9—C10—C11—C8^i^	33.9 (3)
C2—N1—C4—C5	−62.9 (3)	C11^i^—C8—C9—Ir1	102.9 (2)
C3—N2—C1—Ir1	179.27 (17)	C11^i^—C8—C9—C10	0.8 (4)
C3—N2—C1—N1	−0.7 (3)		

Symmetry code: (i) −*x*+3/2, −*y*+3/2, *z*.
